# Anti-inflammatory effects of heat-killed *Lactobacillus plantarum* L-137 on cardiac and adipose tissue in rats with metabolic syndrome

**DOI:** 10.1038/s41598-018-26588-x

**Published:** 2018-05-25

**Authors:** Ayako Uchinaka, Naoki Azuma, Hisashi Mizumoto, Shiho Nakano, Moeko Minamiya, Mamoru Yoneda, Kiyoshi Aoyama, Yuki Komatsu, Yuichiro Yamada, Toyoaki Murohara, Kohzo Nagata

**Affiliations:** 1 Department of Pathophysiological Laboratory Sciences, Nagoya University Graduate School of Medicine, Nagoya, Japan; 2 Department of Medical Technology, Nagoya University School of Health Sciences, Nagoya, Japan; 3 Department of Cardiology, Nagoya University Graduate School of Medicine, Nagoya, Japan

## Abstract

The effects of heat-killed *Lactobacillus plantarum* L-137 (HK L-137) on chronic inflammation associated with metabolic disorders have remained unknown. We examined the effects of HK L-137 on cardiac and adipose tissue pathophysiology in DahlS.Z-*Lepr*^*fa*^/*Lepr*^*fa*^ (DS/obese) rats as a model of metabolic syndrome. DS/obese rats were treated orally with HK L-137 (2 or 75 mg kg^−1^ day^−1^) from 9 to 13 weeks of age. HK L-137 attenuated left ventricular (LV) inflammation and fibrosis as well as adipocyte hypertrophy, inflammation, and up-regulation of sterol regulatory element–binding protein–1c (SREBP-1c) gene expression in visceral and subcutaneous adipose tissue, without affecting body weight gain or hypertension. The low dose of HK L-137 also ameliorated LV diastolic dysfunction, the increase in subcutaneous fat mass, and insulin resistance as well as attenuated the down-regulation of Akt phosphorylation in visceral and subcutaneous adipose tissue, and the elevation of the circulating interleukin-6 concentration. Furthermore, the proportion of regulatory T (Treg) cells among CD4^+^ T cells in the spleen was increased by HK L-137. These results suggest that the anti-inflammatory effects of HK L-137 on the heart and adipose tissue are related, at least partly, to suppression of systemic inflammation associated with an increase in splenic Treg cell.

## Introduction

The role of the gut microbiome in human health and pathological conditions such as obesity and metabolic syndrome (MetS) has recently attracted much attention^1,2^. MetS and other chronic diseases thus result in part from complex interactions among enterocytes, immunocytes, and gut bacteria^3^. Chronic inflammation underlies various slowly disabling disorders, and disruption in the balance of the normal intestinal bacterial flora triggers aberrations in the immune system^3^. Probiotics consist of live microorganisms that confer health benefits to the host, whereas prebiotics are nondigestible food ingredients that also confer such benefit^4–7^. In addition, nonviable microbes have shown beneficial effects equivalent to, or even greater than, those of live microbes^8^. Microorganisms that promote health by influencing immune mechanisms in gut-associated lymphoid tissue (GALT), which is a major site of host encounter with exogenous antigens and pathogens^9^, have been termed “immunobiotics”^10^. These latter organisms can affect the composition of the gut microbiota and thereby reduce the production of proinflammatory factors by bacteria and improve barrier integrity. It is also recognized that the interaction of GALT with microbiota regulates both the quality and quantity of systemic immune response.

Several lactic acid bacteria are administered as probiotics or immunobiotics because of their immune-enhancing effects^11,12^. Heat-killed *Lactobacillus plantarum* L-137 (HK L-137), a bacterial strain isolated from a fermented fish and rice dish^13^, induces the production of interleukin (IL)–12 by macrophages and dendritic cells^14,15^ and thereby promotes activation of T helper (Th) 1–related immune responses^16,17^. HK L-137 exerts antiallergic^18^ and antitumor^14^ effects as well as protects against influenza virus infection^19^ in humans or mouse models. However, the effects of HK L-137 on chronic inflammation associated with metabolic disorders have remained unclear.

We have established the DahlS.Z-*Lepr*^*fa*^/*Lepr*^*fa*^ (DS/obese) rat, derived from a cross between Dahl salt-sensitive and Zucker rats, as an animal model of MetS. These animals develop salt-sensitive hypertension as well as left ventricular (LV) diastolic dysfunction, hypertrophy, and fibrosis^20^, and these conditions are accompanied by increased cardiac oxidative stress and inflammation^21^. We have now examined the effects of HK L-137 on cardiac and adipose tissue pathology associated with MetS in DS/obese rats.

## Results

### Physiological data

**Figure 1:**
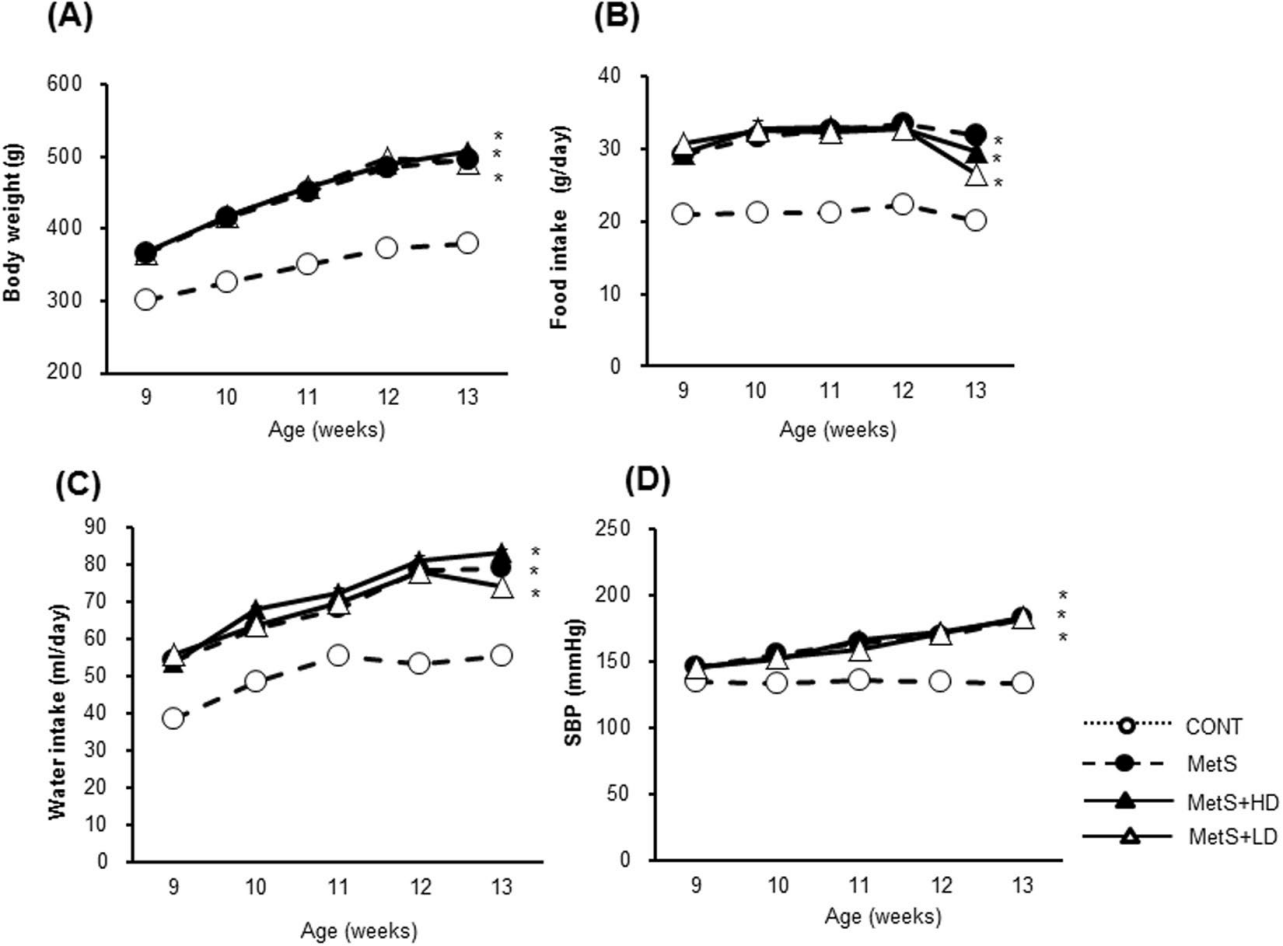
Time courses of body weight, food and water intake, and SBP in rats of the four experimental groups. Body weight (**A**), food (**B**) and water (**C**) intake, and SBP (**D**) were measured in rats at the indicated ages. Data are means ± SEM (*n* = 8, 8, 12, and 12 for CONT, MetS, MetS + HD, and MetS + LD groups, respectively). *P < 0.05 versus CONT.

To investigate the effects of HK L-137 on physiological parameters in MetS rats, we evaluated body weight (Fig. 1A), food and water intake (Fig. 1B,C), and systolic blood pressure (SBP) (Fig. 1D). All of these parameters were significantly higher in the MetS group than in the CONT group, and these differences were not affected by HK L-137. At 13 weeks of age, the ratios of heart or LV weight to tibial length—indicators of cardiac and LV hypertrophy, respectively—were significantly increased in the MetS group compared with the CONT group, and these increases were not affected by HK L-137 (Table 1). Furthermore, the ratios of liver, kidney, visceral (retroperitoneal, epididymal, or mesenteric) fat, or interscapular brown adipose tissue (BAT) weight to tibial length were also greater in the MetS group than in the CONT group, and these differences were not influenced by HK L-137. In contrast, low-dose HK L-137 resulted in a significant attenuation of the increase in the ratio of subcutaneous (inguinal) fat weight to tibial length apparent in the MetS group.

**Table 1:** Physiological, morphological, and cardiac functional parameters for rats of the four experimental groups at 13 weeks of age.

	CONT	MetS	MetS + HD	MetS + LD
**Physiological parameters**				
Heart weight/tibial length (mg/mm)	38.22 ± 1.87	49.20 ± 2.23*	51.26 ± 1.90*	50.62 ± 2.25*
LV weight/tibial length (mg/mm)	28.12 ± 1.56	37.35 ± 1.78*	38.37 ± 1.38*	37.62 ± 1.46*
Liver weight/tibial length (mg/mm)	330.86 ± 22.26	748.82 ± 65.55*	680.56 ± 37.51*	681.43 ± 51.77*
Kidney weight/tibial length (mg/mm)	92.22 ± 3.89	125.02 ± 9.64*	125.83 ± 5.96*	124.89 ± 8.33*
Retroperitoneal fat weight/tibial length (mg/mm)	95.25 ± 4.50	413.10 ± 13.16*	428.62 ± 10.86*	414.07 ± 12.30*
Epididymal fat weight/tibial length (mg/mm)	111.98 ± 3.44	342.60 ± 14.57*	340.38 ± 9.58*	322.79 ± 9.87*
Mesenteric fat weight/tibial length (mg/mm)	83.05 ± 2.83	368.94 ± 10.44*	359.74 ± 9.87*	374.26 ± 10.95*
Inguinal fat weight/tibial length (mg/mm)	216.61 ± 4.95	1306.00 ± 43.85*	1345.78 ± 64.76*	1136.88 ± 61.09 *†‡
BAT weight/tibial length (mg/mm)	10.41 ± 0.72	26.10 ± 1.84*	23.96 ± 1.53*	26.07 ± 1.38*
**Cardiac functional parameters**				
IVST (mm)	1.48 ± 0.02	1.88 ± 0.05*	1.88 ± 0.03*	1.89 ± 0.02*
LVDd (mm)	8.11 ± 0.15	8.19 ± 0.16	8.29 ± 0.090	8.15 ± 0.13
LVPWT (mm)	1.45 ± 0.019	1.91 ± 0.038*	1.83 ± 0.038*	1.88 ± 0.011*
LV mass (mg)	776.78 ± 25.55	1054.66 ± 48.55*	1021.44 ± 30.34*	1028.73 ± 18.28*
RWT	0.36 ± 0.0060	0.46 ± 0.0010*	0.45 ± 0.0093*	0.47 ± 0.0092*
LVFS (%)	31.39 ± 0.94	43.80 ± 2.31*	47.61 ± 1.32*	44.26 ± 1.42*
LVEF (%)	67.34 ± 1.25	81.23 ± 2.06*	85.05 ± 1.07*	82.13 ± 1.33*
E/A	1.97 ± 0.09	1.54 ± 0.06*	1.52 ± 0.06*	1.55 ± 0.09*
DcT (ms)	41.40 ± 1.50	48.98 ± 1.91*	47.05 ± 1.03*	41.36 ± 0.76^†,‡^
IRT (ms)	31.16 ± 1.93	36.2 ± 1.53*	32.83 ± 1.41	30.63 ± 1.71^†,‡^
Tau (ms)	20.21 ± 0.25	29.11 ± 0.26*	25.30 ± 1.74*	23.78 ± 1.05
LVEDP (mmHg)	4.56 ± 0.32	9.80 ± 1.43*	7.94 ± 0.48*	5.57 ± 0.33^†,‡^
LVEDP/LVDd (mmHg/mm)	0.56 ± 0.04	1.17 ± 0.19*	0.96 ± 0.06*	0.66 ± 0.05^†^

Data are means ± SEM (organ weight and echocardiography, *n* = 8, 8, 12, and 12; cardiac catheterization, *n* = 4, 4, 6, and 6 for DS/lean rats treated with vehicle [CONT] or DS/obese rats treated with vehicle [MetS], a high dose of HK L-137 [MetS + HD], or a low dose of HK L-137 [MetS + LD], respectively). *P < 0.05 versus CONT, ^†^*P* < 0.05 versus MetS, ^‡^*P* < 0.05 versus MetS + HD.

### Cardiac function

We performed echocardiography and cardiac catheterization to assess the effects of HK L-137 on cardiac morphology and LV systolic and diastolic function in Mets rats (Table 1). Echocardiography revealed that rats in the MetS group had a greater interventricular septum (IVST) and LV posterior wall (LVPWT) thickness, LV mass, relative wall thickness (RWT), LV fractional shortening (LVFS), and LV ejection fraction (LVEF) relative to the CONT group (Table 1). Treatment of DS/obese rats with the high or low dose of HK L-137 had no significant effects on these differences. The ratio of early to late ventricular filling velocities (E/A) was decreased in the MetS group in a manner insensitive to HK L-137. The deceleration time (DcT), isovolumic relaxation time (IRT), and tau—all of which are indices of LV relaxation—as well as LV end-diastolic pressure (LVEDP) determined by cardiac catheterization and the ratio of LVEDP to LV end-diastolic dimension (LVDd), an index of LV diastolic stiffness, were all increased in the MetS group compared with the CONT group. The changes in DcT, IRT, LVEDP, and the ratio of LVEDP to LVDd were significantly attenuated in the MetS + LD group compared with the MetS group. Tau also tended to be reduced in the MetS + LD group compared with the MetS group (*P* = 0.07). Together, these data thus indicated that the low dose of HK L-137 was protective against LV diastolic dysfunction in DS/obese rats.

### Lipid metabolism

To determine whether HK L-137 affects lipid metabolism in MetS rats, we examined serum lipid profiles. The fasting serum levels of total cholesterol, low-density lipoprotein (LDL)-cholesterol, and triglyceride were increased in MetS rats relative to CONT rats, and these differences were not affected by HK L-137 (Table 2). The high-density lipoprotein (HDL)-cholesterol level was also increased in the MetS group compared with the CONT group, and this effect was attenuated by the high dose but not the low dose of HK L-137. Free fatty acid levels tended to be increased in the MetS group (*P* = 0.08) and were significantly elevated in both MetS + HD and MetS + LD groups relative to the CONT group.

**Table 2:** Metabolic parameters of rats in the four experimental groups at 13 weeks of age.

Parameter	CONT	MetS	MetS + HD	MetS + LD
Total cholesterol (mg/dl)	71.00 ± 6.51	380.50 ± 49.92*	356.33 ± 37.69*	351.17 ± 58.17*
LDL-cholesterol (mg/dl)	14.40 ± 2.20	73.75 ± 10.35*	77.83 ± 14.34*	73.33 ± 23.85*
HDL-cholesterol (mg/dl)	18.80 ± 1.16	47.50 ± 3.38*	40.67 ± 0.88*^,†^	43.20 ± 2.65*
Triglyceride (mg/dl)	80.20 ± 4.08	2442.25 ± 392.19*	2369.50 ± 545.16*	1862.13 ± 433.61*
Free fatty acids (mEq/l)	0.69 ± 0.08	0.99 ± 0.03	1.15 ± 0.11*	1.16 ± 0.13*
Urinary protein (mg/dl)	690.28 ± 41.62	1378.41 ± 181.17*	1324.45 ± 105.97*	1566.74 ± 90.04*
Serum creatinine (mg/dl)	0.31 ± 0.01	0.85 ± 0.10*	0.92 ± 0.14*	0.76 ± 0.18*
Creatinine clearance (ml min^−1^ 100 g^−1^ body weight)	0.61 ± 0.076	0.14 ± 0.031*	0.17 ± 0.03*	0.21 ± 0.044*
Creatinine clearance (ml min^−1^ g^−1^ kidney weight)	0.60 ± 0.105	0.17 ± 0.052*	0.18 ± 0.039*	0.24 ± 0.065*
Serum IL-6 (pg/ml)	33.85 ± 7.22	123.81 ± 6.24*	101.19 ± 6.24*	101.22 ± 5.38*^,†^
Serum IL-1β (pg/ml)	55.25 ± 8.99	198.75 ± 46.28*	136.12 ± 33.1*	59.4 ± 29.28^†^

Data are means ± SEM (*n* = 4, 4, 6, and 6 for CONT, MetS, MetS + HD, and MetS + LD groups, respectively). *P < 0.05 versus CONT, ^†^*P* < 0.05 versus MetS.

### Renal function

To determine the effects of HK L-137 on renal function in MetS rats, we performed blood and urine tests. Urinary protein and serum creatinine levels were increased whereas creatinine clearance was decreased in the MetS group compared with the CONT group in a manner insensitive to HK L-137 (Table 2).

### LV pathology

**Figure 2:**
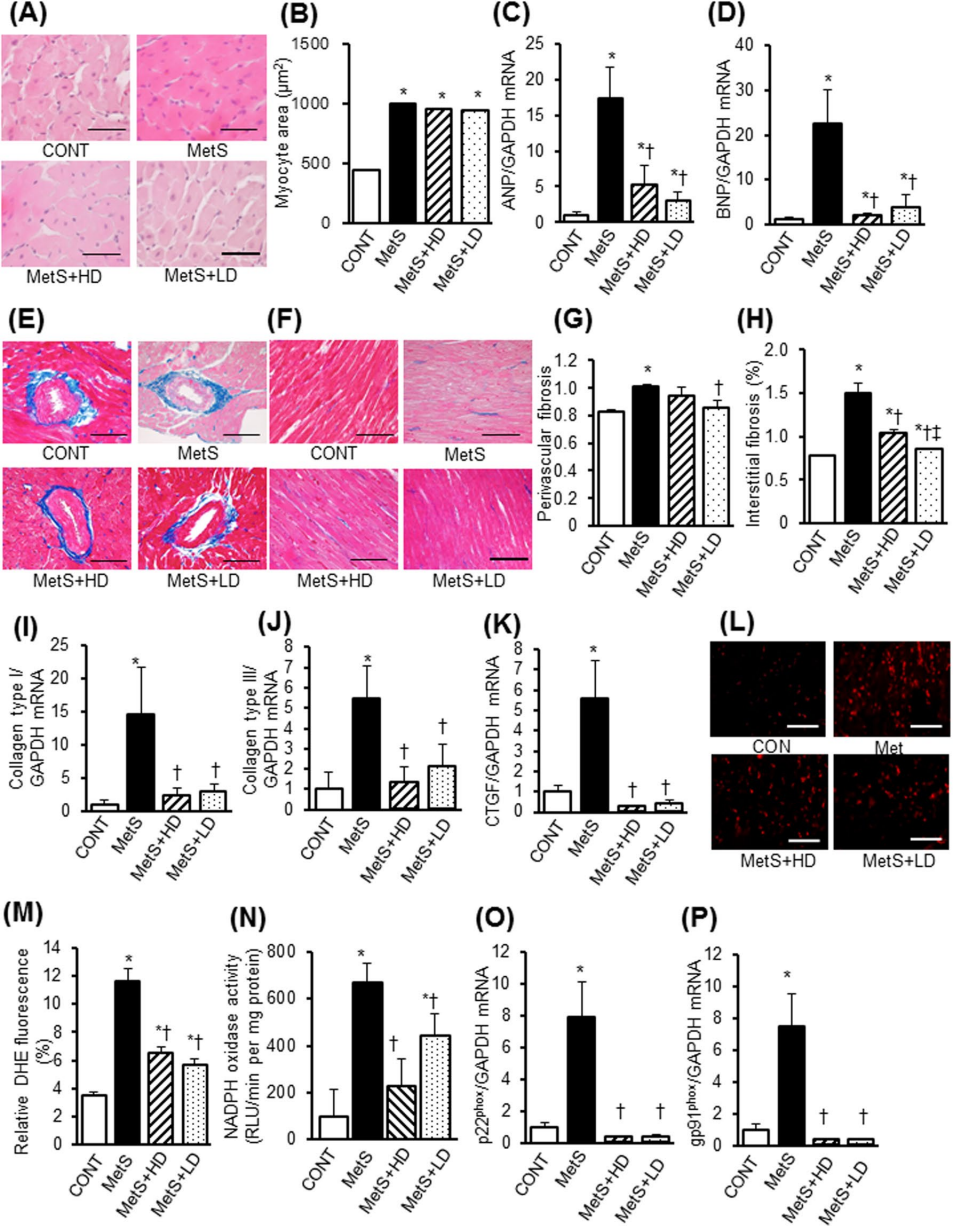
Effects of HK L-137 on cardiac injury. (**A**) Representative H&E staining of LV tissue. Bars, 100 μm. (**B**) Myocyte surface area determined from sections as in (**A**). (**C**,**D**) Quantitative RT-PCR analysis of relative ANP (**C**) and BNP (**D**) mRNA abundance in the left ventricle. (**E**,**F**) Representative perivascular (**E**) and interstitial (**F**) fibrosis in the left ventricle revealed by Azan-Mallory staining. Bars, 50 μm. (**G**,**H**) Quantification of cardiac fibrosis in sections similar to those in (**E**) and (**F**). (**I**–**K**) Quantitative RT-PCR analysis of relative collagen type I (**I**), collagen type III (**J**), and CTGF (**K**) mRNA abundance in the left ventricle. (**L**) Representative confocal fluorescence microscopy of frozen LV tissue stained with DHE. Bars, 50 µm. (**M**) Quantification of relative DHE fluorescence in sections similar to those in (**A**). (**N**) NADPH oxidase activity in LV extracts. RLU, relative light units. (**O**,**P**) Quantitative RT-PCR analysis of relative p22^phox^ (**O**) and gp91^phox^ (**P**) mRNA abundance in the left ventricle. All quantitative data are means ± SEM (*n* = 8, 8, 12, and 12 [**B**,**G**,**H** and **L**–**N**] or *n* = 5, 5, 7, and 7 [**C**,**D**,**I**–**K**,**O** and **P**] for CONT, MetS, MetS + HD, and MetS + LD groups, respectively). *P < 0.05 versus CONT, ^†^*P* < 0.05 versus MetS, ^‡^*P* < 0.05 versus MetS + HD.

We examined the effects of HK L-137 on LV injury and oxidative stress. The increase in cardiomyocyte cross-sectional area apparent in the MetS group compared with the CONT group was not affected by HK L-137 (Fig. 2A,B). In contrast, the increased expression of atrial natriuretic peptide (ANP) and brain natriuretic peptide (BNP) genes also apparent in the heart of DS/obese rats was attenuated by HK L-137 at both doses (Fig. 2C,D). The deposition of collagen in LV perivascular (Fig. 2E,G) and interstitial (Fig. 2F,H) regions was significantly increased in the MetS group compared with the CONT group, with the former effect being inhibited by the low dose of HK L-137 and the latter effect by both doses. Furthermore, interstitial fibrosis was inhibited to a greater extent in the MetS + LD group than in the MetS + HD group. Compared with the CONT group, the amounts of collagen type I and III and connective tissue growth factor (CTGF) mRNAs were increased in the MetS group, and these effects were prevented by HK L-137 at either dose (Fig. 2I–K).

**Figure 3:**
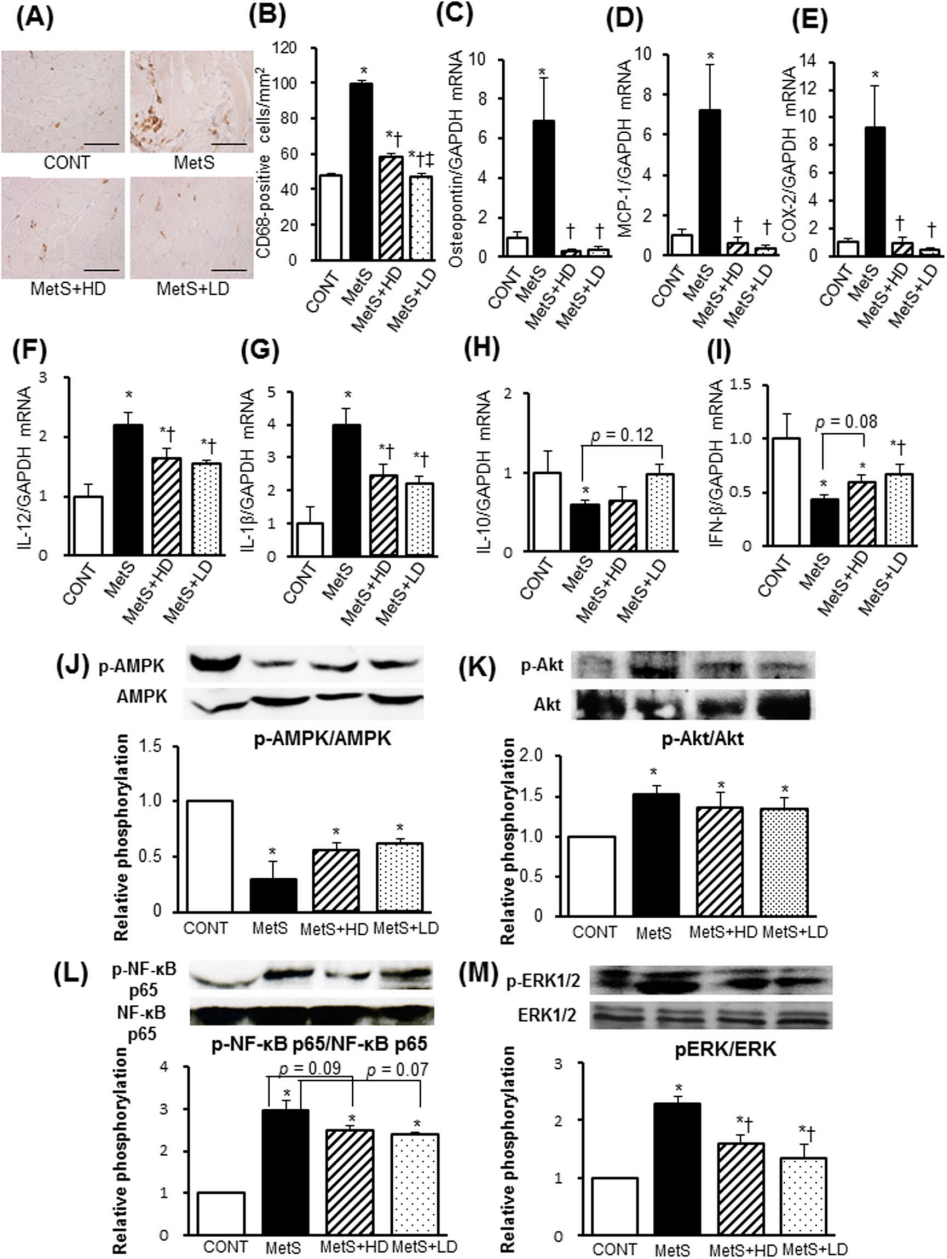
Effects of HK L-137 on LV inflammation and insulin signaling. (**A**) Representative immunostaining of CD68 for detection of macrophages in LV tissue. Bars, 100 µm. (**B**) Quantification of CD68^+^ cell density in sections similar to those in (**A**). (**C**–**I**) Quantitative RT-PCR analysis of relative osteopontin (**C**), MCP-1 (**D**), COX-2 (**E**), IL-12 (**F**), IL-1β (**G**), IL-10 (**H**), and IFN-β (**I**) mRNA abundance in the left ventricle. (**J**–**M**) Representative immunoblot analysis and densitometric quantification of phosphorylated (p−) and total forms of AMPK (**J**), Akt (**K**), NF-κB p65 (**L**), and ERK1/2 (**M**) in the left ventricle. Data are means ± SEM (*n* = 8, 8, 12, and 12 [**B**] or *n* = 5, 5, 7, and 7 [**C**–**M**] for CONT, MetS, MetS + HD, and MetS + LD groups, respectively). *P < 0.05 versus CONT, ^†^*P* < 0.05 versus MetS.

LV oxidative stress was increased in MetS rats, as indicated by increased superoxide production (Fig. 2L,M) and NADPH oxidase activity (Fig. 2N), and these effects were suppressed by HK L-137 treatment. The up-regulation of p22^phox^ and gp91^phox^ mRNAs apparent in the left ventricle of MetS rats was also prevented by HK L-137 at either dose (Fig. 2O,P). Immunohistochemical staining of LV tissue for CD68 in order to detect cells of the monocyte-macrophage lineage revealed that the extent of macrophage infiltration was significantly increased in the MetS group in a manner sensitive to HK L-137 at either dose, with the effect of the low dose being greater than that of the high dose (Fig. 3A,B). The up-regulation of osteopontin, monocyte chemoattractant protein–1 (MCP-1), and cyclooxygenase-2 (COX-2) mRNAs in the MetS group was blocked by HK L-137 at either dose (Fig. 3C–E). Furthermore, the obesity-induced upregulation of IL-12 and IL-1β genes were also suppressed by both doses of HK L-137 (Fig. 3F,G). In contrast, the amount of IL-10 mRNA was reduced in the MetS group, and this reduction tended to be attenuated in the MetS + LD group (*P* = 0.12, Fig. 3H). The reduction of interferon (IFN)-β gene expression in the MetS group was attenuated in the MetS + LD group (Fig. 3I) and tended to be reversed in the MetS + HD group (*P* = 0.08). The phosphorylation (activity) of AMP-activated protein kinase (AMPK) was reduced and that of Akt increased in the heart of MetS rats in a manner insensitive to HK L-137 (Fig. 3J,K). In the meanwhile, HK L-137 exhibited a tendency to attenuate the increase in the amount of phosphorylated form of p65 subunit of nuclear factor-kappa B (NF-κB) in the MetS group (*P* = 0.09 versus MetS + HD, *P* = 0.07 versus MetS + LD; Fig. 3L). The increase in the phosphorylation of extracellular signal-regulated kinase (ERK)1/2 in the metS group was also attenuated by HK L-137 at both doses (Fig. 3M).

### Visceral adipose tissue pathology

**Figure 4:**
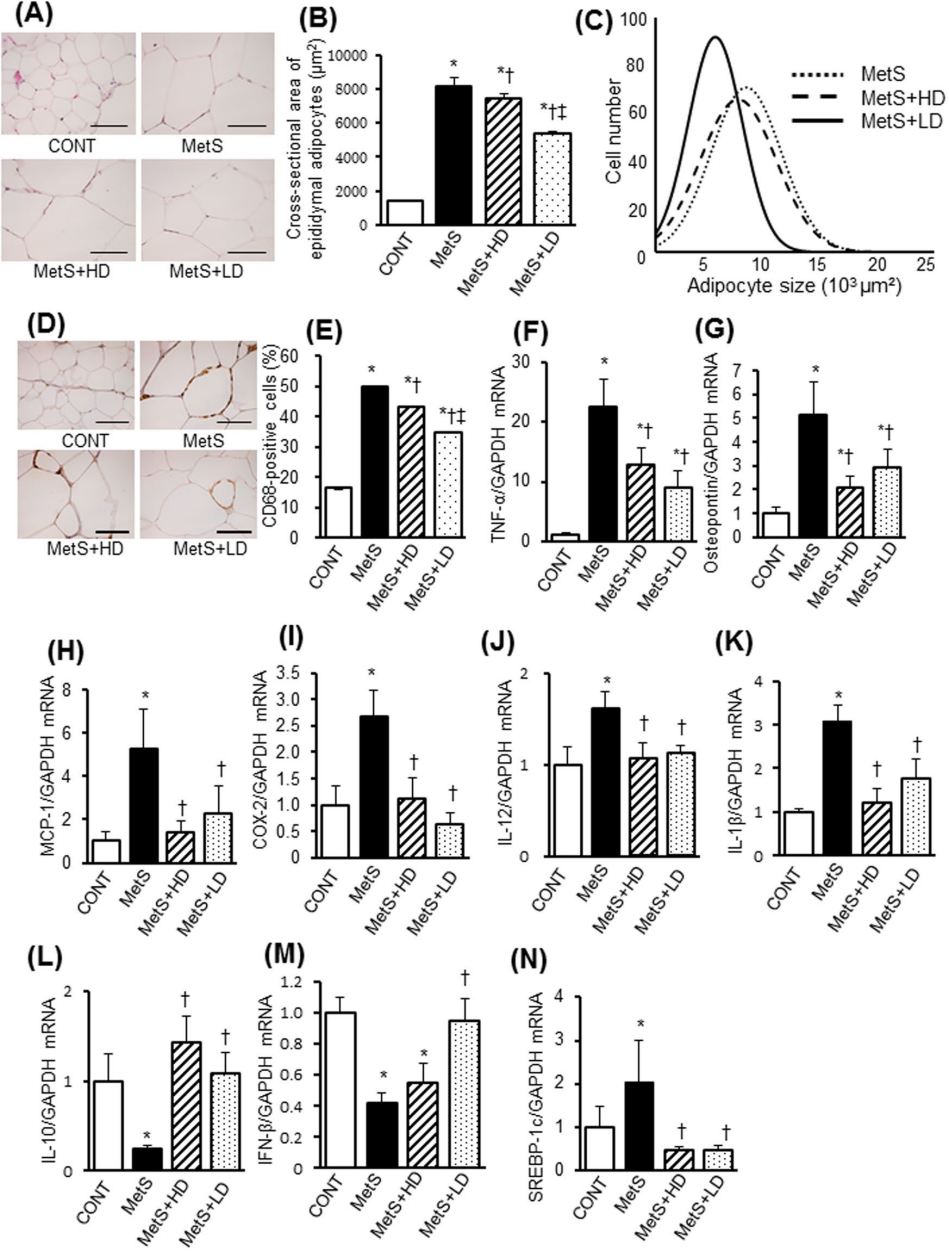
Effects of HK L-137 on visceral adipose tissue pathology. (**A**) Representative H&E staining of epididymal adipose tissue sections. Bars, 100 µm. (**B**,**C**) Adipocyte cross-sectional area (**B**) and distribution of adipocyte size (**C**) determined from sections as in (**A**). (**D**) Representative immunostaining of CD68 for detection of macrophages in epididymal adipose tissue. Bars, 100 µm. (**E**) Number of nuclei for CD68-positive cells as a percentage of total nuclei determined from sections as in (**D**). (**F**–**N**) Quantitative RT-PCR analysis of relative TNF-α (**F**), osteopontin (**G**), MCP-1 (**H**), COX-2 (**I**), IL-12 (**J**), IL-1β (**K**), IL-10 (**L**), IFN-β (**M**), and SREBP-1c (**N**) mRNA abundance in epididymal adipose tissue. All quantitative data with the exception of those in (**C**) are means ± SEM (*n* = 8, 8, 12, and 12 [**B**,**C** and **E**] or *n* = 5, 5, 7, and 7 [**F**–**N**] for CONT, MetS, MetS + HD, and MetS + LD groups, respectively). *P < 0.05 versus CONT, ^†^*P* < 0.05 versus MetS, ^‡^*P* < 0.05 versus MetS + HD.

**Figure 5:**
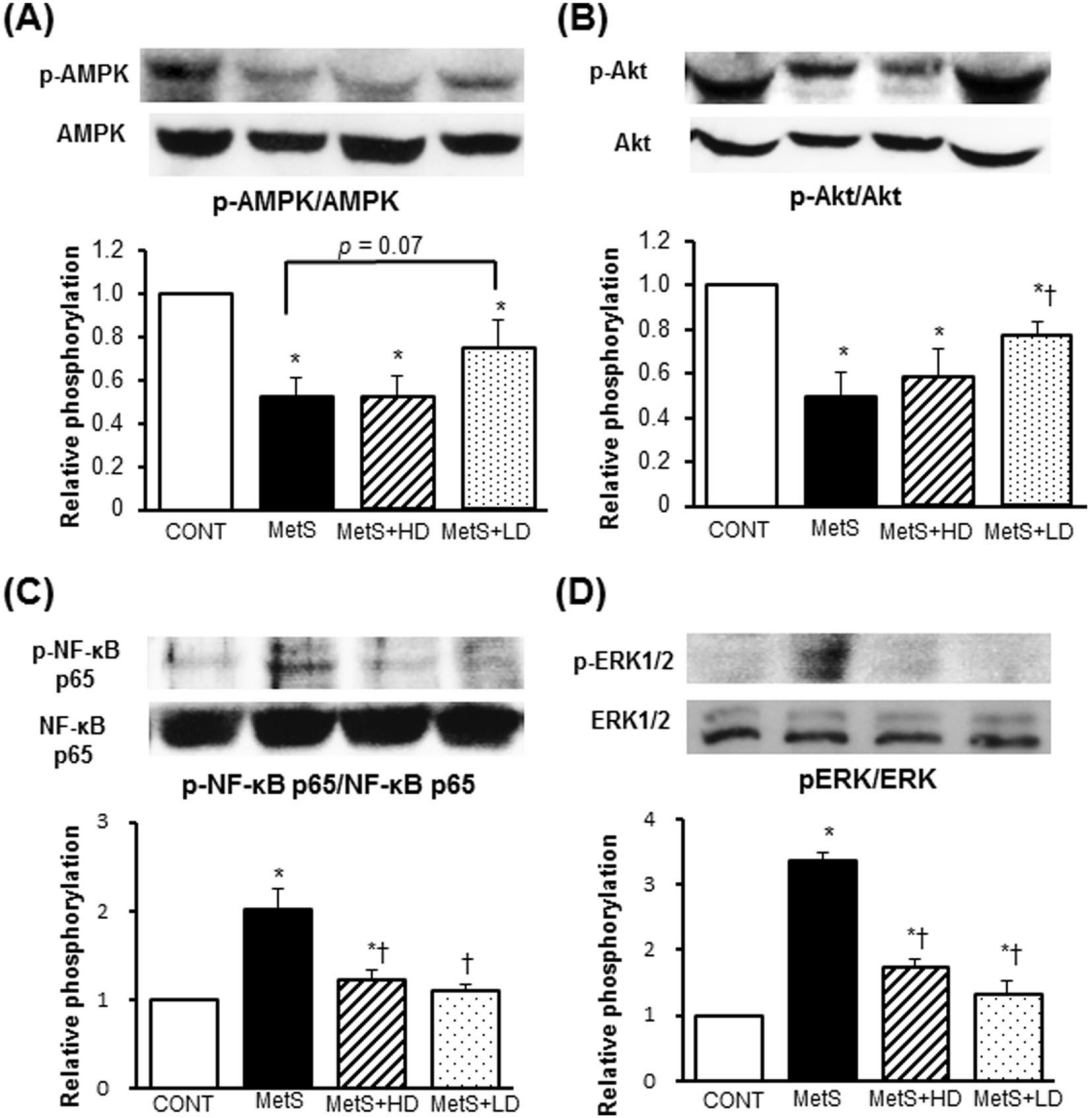
Effects of HK L-137 on insulin signaling in epididymal adipose tissue pathology. (**A**–**D**) Representative immunoblot analysis and densitometric quantification of phosphorylated (p−) and total forms of AMPK (**A**), Akt (**B**), NF-κB p65 (**C**), and ERK1/2 (**D**) in epididymal adipose tissue. All quantitative data are means ± SEM (*n* = 5, 5, 7, and 7 for CONT, MetS, MetS + HD, and MetS + LD groups, respectively). *P < 0.05 versus CONT, ^†^*P* < 0.05 versus MetS, ^‡^*P* < 0.05 versus MetS + HD.

Since adipose tissue inflammation plays an important role in obesity-induced insulin resistance, we examined the effects of HK L-137 on visceral adipose tissue pathology and cytokine gene expression. The size of adipocytes in epididymal adipose tissue was larger in the MetS group than in the CONT group, and this difference was attenuated by HK L-137 at the low dose to a greater extent than at the high dose (Fig. 4A,B). Analysis of adipocyte size distribution also revealed that, compared with the MetS group, the low dose of HK L-137 induced a more pronounced shift toward smaller cells than did the high dose (Fig. 4C). The low dose of HK L-137 attenuated the obesity-associated infiltration of macrophages in epididymal fat tissue more effectively than did the high dose (Fig. 4D,E). The up-regulation of tumor necrosis factor–α (TNF-α), osteopontin, MCP-1, COX-2, IL-12, IL-1β and SREBP-1c mRNAs in this tissue of DS/obese rats was similarly attenuated by HK L-137 at either dose (Fig. 4F–K and N). HK L-137 also attenuated the down-regulation of IL-10 gene expression in DS/obese rats (Fig. 4L). The decrease in the level of IFN-β gene expression in the MetS group was completely prevented in the MetS + LD group but was unaltered in the MetS + HD group (Fig. 4M). AMPK activity was reduced in visceral adipose tissue of the MetS group, and this effect tended to be attenuated in the MetS + LD group (*P* = 0.07, Fig. 5A). DS/obese rats also manifested a reduction in Akt activity in epididymal adipose tissue, and this effect was significantly inhibited by the low dose of HK L-137 (Fig. 5B). Furthermore, increased phosphorylations of the p65 subunit of NF-κB and ERK1/2 in MetS rats were suppressed by HK L-137 at either dose (Fig. 5C,D).

### Subcutaneous adipose tissue pathology

**Figure 6:**
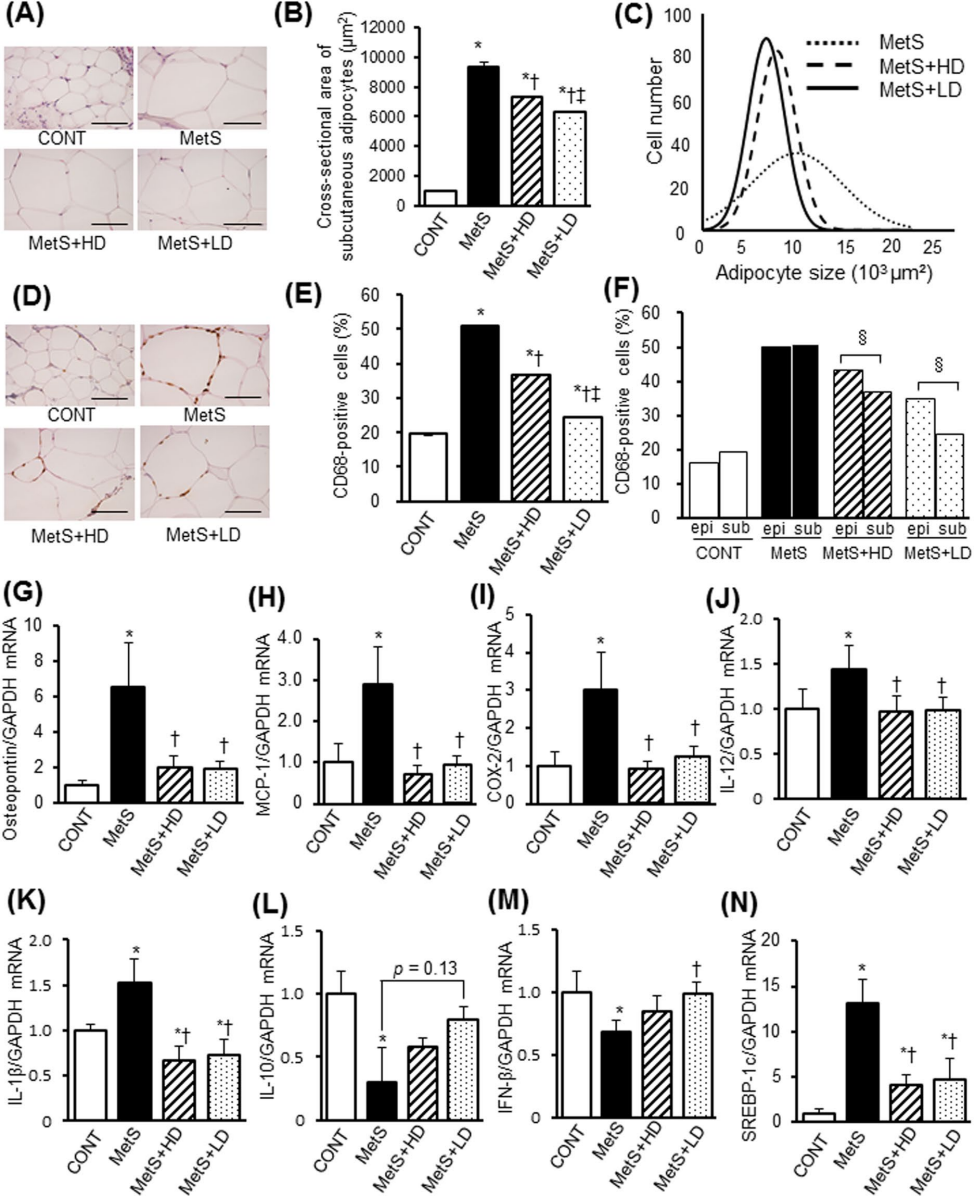
Effects of HK L-137 on subcutaneous adipose tissue pathology. (**A**) Representative H&E staining of inguinal adipose tissue sections. Bars, 100 µm. (**B**,**C**) Adipocyte cross-sectional area (**B**) and distribution of adipocyte size (**C**) determined from sections as in (**A**). (**D**) Representative immunostaining of CD68 for detection of macrophages in inguinal adipose tissue. Bars, 100 µm. (**E**) Number of nuclei for CD68-positive cells as a percentage of total nuclei determined from sections as in (**D**). (**F**) Comparison of macrophage infiltration between epididymal (epi) (Fig. 4E) and subcutaneous (sub) (**E**) adipose tissue. (**G**–**N**) Quantitative RT-PCR analysis of relative osteopontin (**G**), MCP-1 (**H**), COX-2 (**I**), IL-12 (**J**), IL-1β (**K**), IL-10 (**L**), IFN-β (**M**), and SREBP-1c (**N**), mRNA abundance in inguinal adipose tissue. All quantitative data with the exception of those in (**C**) are means ± SEM (*n* = 8, 8, 12, and 12 [**B**,**C**,**E** and **F**] or *n* = 5, 5, 7, and 7 [**G**–**N**] for CONT, MetS, MetS + HD, and MetS + LD groups, respectively). *P < 0.05 versus CONT, ^†^*P* < 0.05 versus MetS, ^‡^*P* < 0.05 versus MetS + HD, ^§^*P* < 0.05.

**Figure 7:**
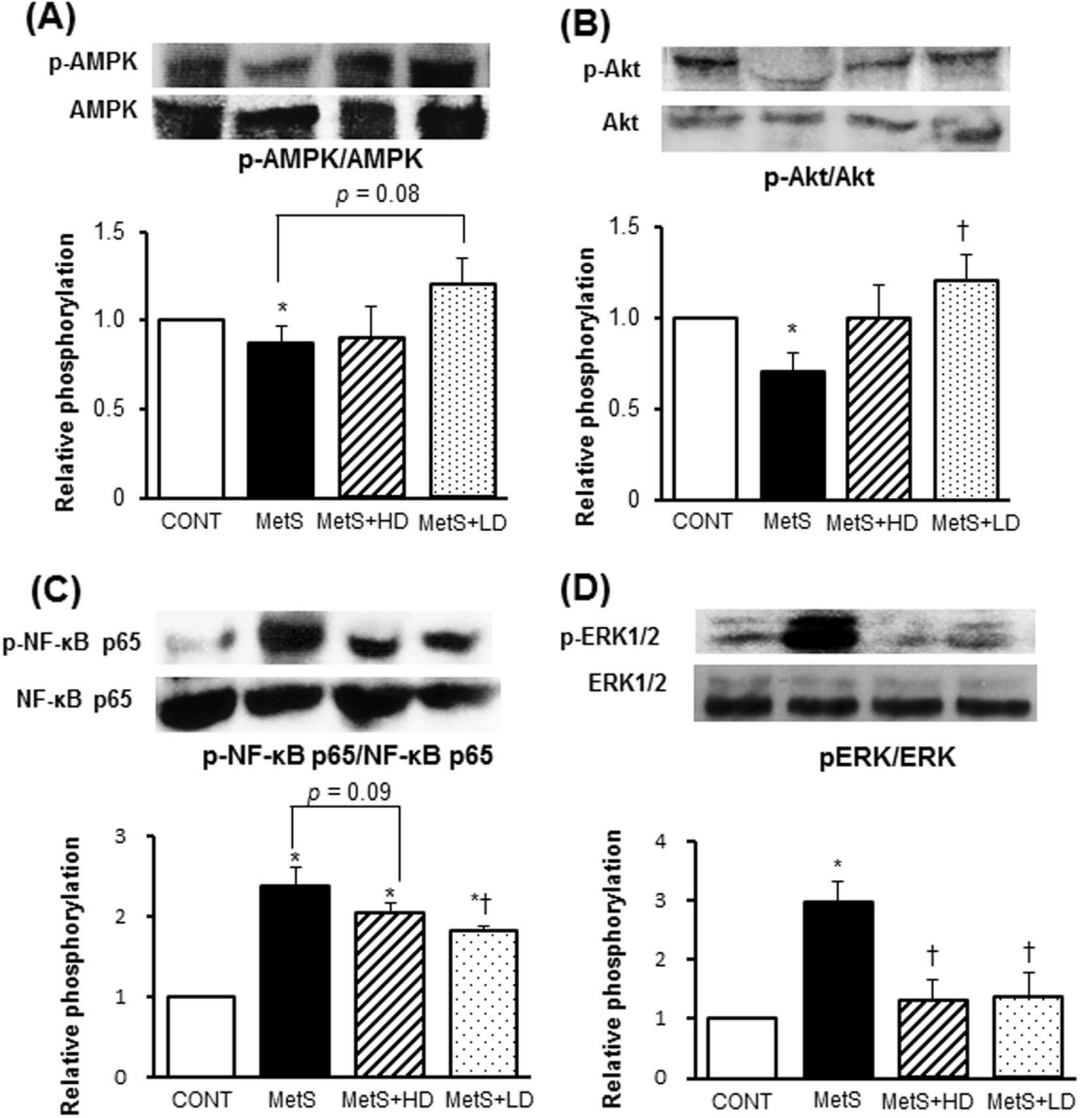
Effects of HK L-137 on insulin signaling in subcutaneous adipose tissue pathology. (**A**–**D**) Representative immunoblot analysis and densitometric quantification of phosphorylated (p−) and total forms of AMPK (**A**), Akt (**B**), NF-κB p65 (**C**), and ERK1/2 (**D**) in inguinal adipose tissue. All quantitative data are means ± SEM (*n* = 5, 5, 7, and 7 for CONT, MetS, MetS + HD, and MetS + LD groups, respectively). *P < 0.05 versus CONT, ^†^*P* < 0.05 versus MetS, ^‡^*P* < 0.05 versus MetS + HD.

We also evaluated the effects of HK L-137 on subcutaneous adipose tissue pathology. The size of adipocytes (Fig. 6A–C) and macrophage infiltration (Fig. 6D,E) were both increased in subcutaneous (inguinal) adipose tissue of the MetS group compared with the CONT group, and these effects were significantly attenuated to a greater extent by the low dose of HK L-137 than by the high dose. Whereas the levels of macrophage infiltration were similar in epididymal and subcutaneous fat tissue of the MetS group, both low and high doses of HK L-137 inhibited macrophage infiltration more effectively in subcutaneous fat than in epididymal fat (Fig. 6F). The increases in osteopontin, MCP-1, COX-2, IL-12, IL-1β and SREBP-1c mRNA levels in subcutaneous adipose tissue of the MetS group were prevented by HK L-137 at either dose (Fig. 6G–K and N). The low dose of HK L-137 also showed a tendency to ameliorate the down-regulation of IL-10 gene expression (*P* = 0.13, Fig. 6L) and prevented the decrease in the abundance of IFN-β mRNA in MetS rats (Fig. 6M). AMPK phosphorylation was reduced in this tissue of the MetS group, with this effect tending to be inhibited by the low dose of HK L-137 (*P* = 0.08, Fig. 7A). The level of Akt phosphorylation was also decreased in the MetS group, and this effect was prevented by the low dose of HK L-137 (Fig. 7B). Furthermore, the up-regulation of NF-κB p65 subunit phosphorylation in MetS rats was suppressed by HK L-137 at low dose (Fig. 7C) and tended to be attenuated by HK L-137 at high dose (*P* = 0.09). The increased activity of ERK1/2 in the MetS group was suppressed by HK L-137 at either dose (Fig. 7D).

### Immunological analysis

**Figure 8:**
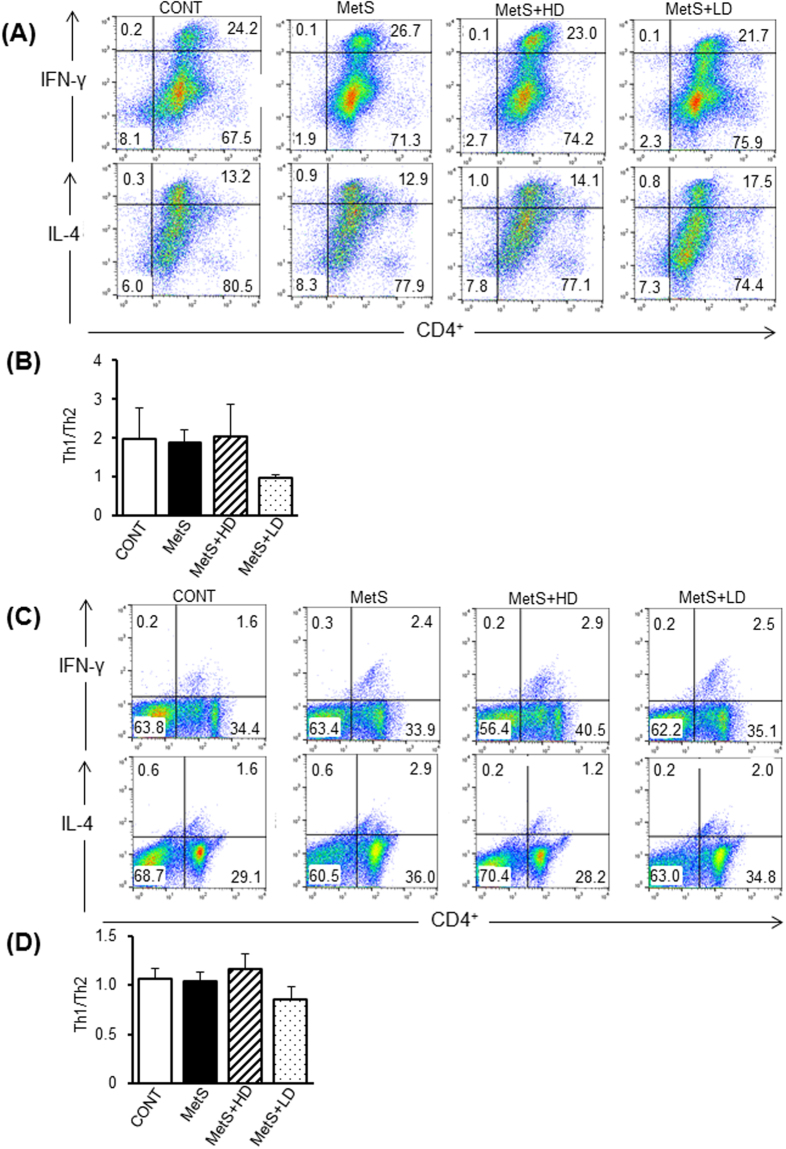
Effects of HK L-137 on the distribution of Th1 and Th2 cells in spleen and epididymal adipose tissue. (**A**) Representative flow cytometric dot plots for the expression of CD4 and IFN-γ or IL-4 in lymphocytes from the spleen. (**B**) The Th1 (CD4^+^IFN-**γ**^**+**^)/Th2 (CD4^+^IL-4^+^) cell ratio in the spleen determined as in (**A**). (**C**) Representative flow cytometric dot plots for the expression of CD4 and IFN-γ or IL-4 in lymphocytes from the epididymal adipose tissue. (**D**) The Th1 (CD4^+^IFN-γ^+^)/Th2 (CD4^+^IL-4^+^) cell ratio in the epididymal adipose tissue determined as in (**C**). Data in (**B** and **D**) are means ± SEM (*n* = 8, 8, 10, and 10 for CONT, MetS, MetS + HD, and MetS + LD groups, respectively).

**Figure 9:**
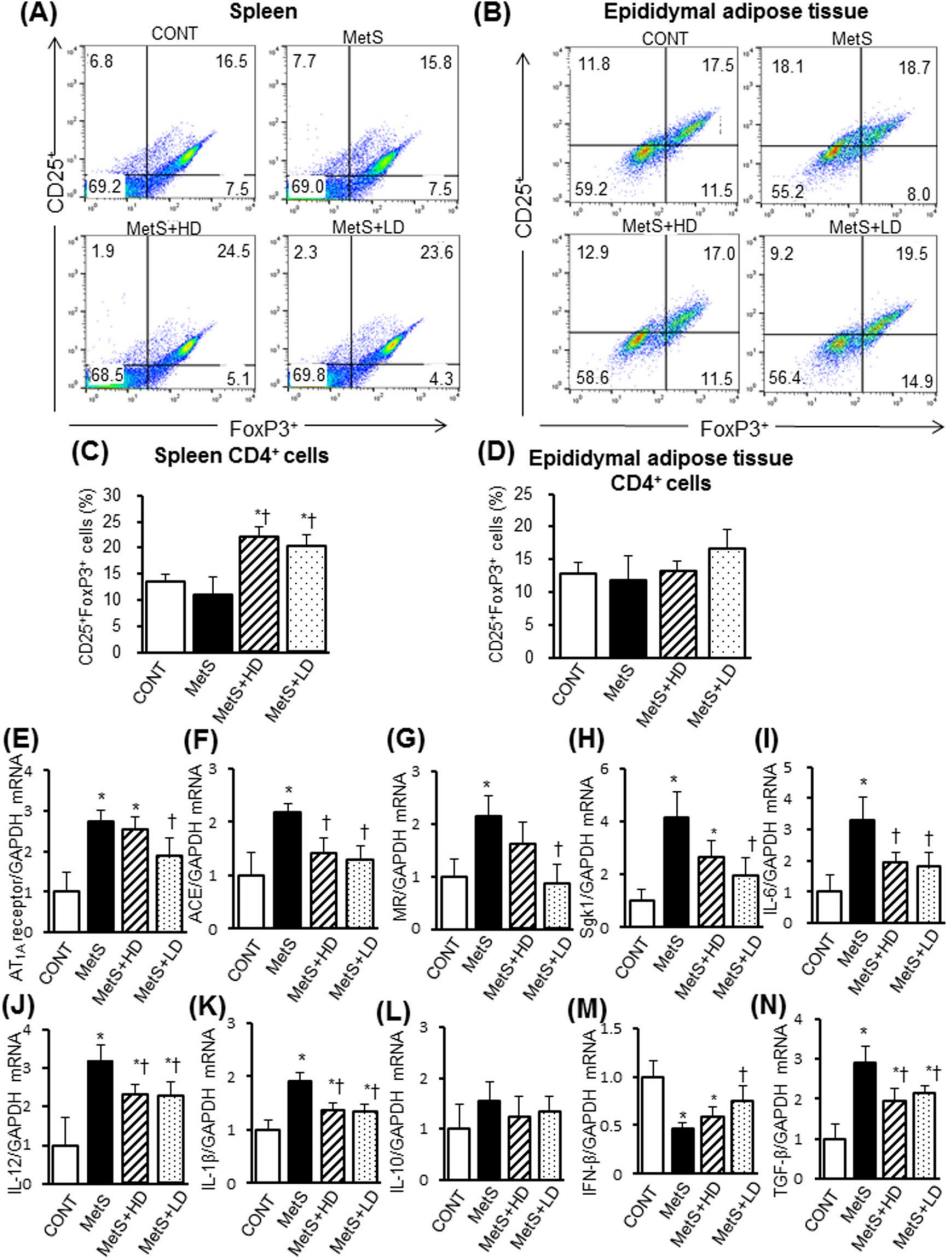
Effects of HK L-137 on the distribution of Treg cells in spleen and epididymal adipose tissue, and RAAS activity and inflammation in spleen. (**A**,**B**) Representative flow cytometric dot plots for the expression of CD25 and Foxp3 in gated CD4^+^ T cells from the spleen (**A**) and epididymal adipose tissue (**B**). (**C**,**D**) Percentage of CD25^+^Foxp3^+^ Treg cells among CD4^+^ T cells from the spleen (**C**) and epididymal adipose tissue (**D**) determined as in (**A** and **B**). (**E**–**N**) Quantitative RT-PCR analysis of relative AT1A receptor (**E**), ACE (**F**), MR (**G**), Sgk1 (**H**), IL-6 (**I**), IL-12 (**J**), IL-1β (**K**), IL-10 (**L**), IFN-β (**M**), and TGF-β (**N**) mRNA abundance in the spleen. Data in (**B**) to (**J**) are means ± SEM (*n* = 8, 8, 12, and 12 [**C** and **D**] or *n* = 5, 5, 7, and 7 [**E**–**N**] for CONT, MetS, MetS + HD, and MetS + LD groups, respectively). *P < 0.05 versus CONT, ^†^*P* < 0.05 versus MetS.

To determine whether HK L-137 induce changes in T cell subsets in the spleen and epididymal adipose tissue, we conducted flow cytometric analysis. The ratio of Th1 to Th2 cells in the spleen (Fig. 8A,B) or epididymal adipose tissue (Fig. 8C,D) did not differ significantly among the four groups of rats. The percentage of CD25^+^Foxp3^+^ regulatory T (Treg) cells among CD4^+^ T cells in the spleen did not differ between MetS and CONT groups but was increased to similar extents by the low or high dose of HK L-137 (Fig. 9A,C). In contrast, the percentage of Treg cells in epididymal adipose tissue was similar for the four experimental groups (Fig. 9B,D). The renin-angiotensin-aldosterone system (RAAS) plays a crucial role in T cell responses during inflammation^22,23^. We thus evaluated expression of RAAS-related and cytokine genes in the spleen. The expression of type 1A receptor for angiotensin II (AT1A receptor), angiotensin-converting enzyme (ACE), mineralocorticoid receptor (MR), and serum/glucocorticoid–regulated kinase 1 (Sgk1) genes in the spleen was increased in the MetS group compared with the CONT group (Fig. 9E–H). These increases were all inhibited by the low dose of HK L-137, whereas those in ACE and Sgkl gene expression were also inhibited by the high dose. The increases in IL-6, IL-12, IL-1β and transforming growth factor-β (TGF-β) mRNA levels in spleen of the MetS group were prevented by HK L-137 at either dose (Fig. 9I–K and N). In contrast, HK L-137 had no effect on the IL-10 expression level in the spleen (Fig. 9L) HK L-137 at low dose also alleviated the reduction in IFN-β mRNA level in the MetS group (Fig. 9M).

### Circulating IL-6 and IL-1β levels

We measured circulating levels of IL-6 and IL-1β to assess the effects of HK L-137 on the extent of systemic inflammation. The MetS group showed a marked increase in the serum concentration of IL-6 and compared with the CONT group (Table 2). This increase was significantly attenuated by the low dose of HK L-137 and tended to be suppressed (*P* = 0.11) by the high dose. The concentration of IL-β level in serum was increased in the MetS group and this effect was also reduced by HK L-137 at low dose (Table 2).

### Glucose metabolism and pancreatic pathology

**Figure 10:**
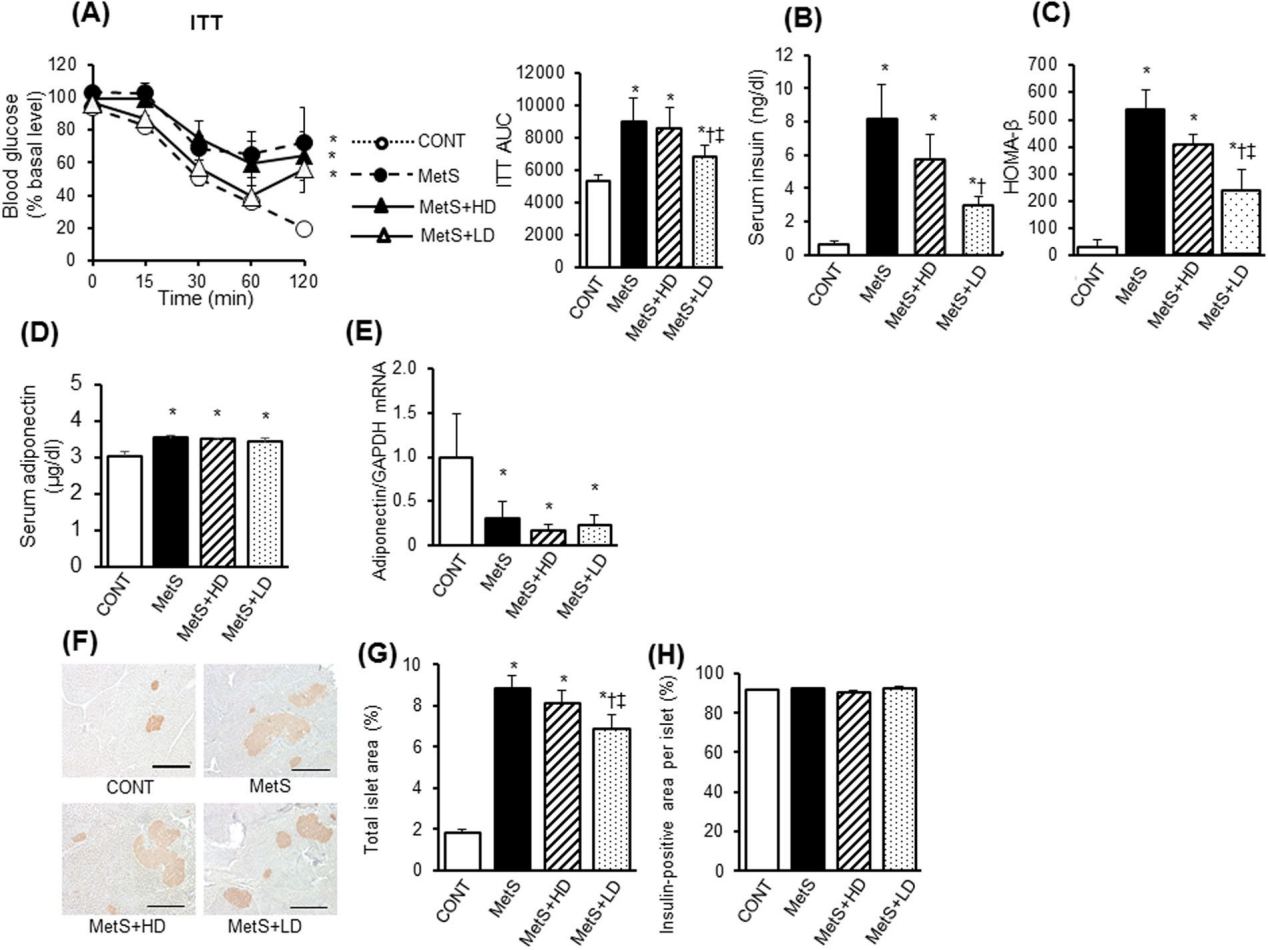
Effects of HK L-137 on glucose homeostasis. (**A**) ITT for rats of the four experimental groups at 13 weeks of age. AUC, area under the curve. (**B**) Fasting serum insulin levels. (**C**) HOMA-β. (**D**) Serum adiponectin levels. (**E**) Quantitative RT-PCR analysis of relative adiponectin mRNA abundance in epididymal adipose tissue. (**F**) Representative immunostaining of insulin in pancreatic sections. Bars, 500 µm. (**G**,**H**) Islet area as a percentage of pancreatic area (**G**) and insulin-positive area per islet (**H**) determined from sections as in (**F**). All quantitative data are means ± SEM (*n* = 5, 5, 6, and 6 [**A**–**C**], *n* = 5, 5, 7, and 7 [**D**,**E**], or *n* = 8, 8, 12, and 12 [**G**,**H**] for the CONT, MetS, MetS + HD, and MetS + LD groups, respectively). *P < 0.05 versus CONT, ^†^*P* < 0.05 versus MetS, ^‡^*P* < 0.05 versus MetS + HD.

To determine whether HK L-137 affects insulin resistance in MetS rats, we performed insulin tolerance test (ITT). An ITT revealed that whole-body insulin sensitivity was impaired in MetS rats, and that this insulin resistance was ameliorated by the low dose of HK L-137 (Fig. 10A). Both the fasting serum insulin level and homeostasis model assessment of β-cell function (HOMA-β) were increased in the MetS group, and these effects were also attenuated by the low dose of HK L-137 (Fig. 10B,C). To explain these changes in glucose metabolism, we also measured circulating adiponectin level and performed immunohistochemistry with pancreatic sections stained for insulin. HK L-137 did not affect either the increase in serum adiponectin concentration or the down-regulation of adiponectin mRNA in epididymal adipose tissue apparent in MetS rats (Fig. 10D,E). The MetS group showed an increase in total islet area per field of pancreatic area compared with the CONT group, and this increase was attenuated by the low dose of HK L-137 (Fig. 10F,G). Finally, the insulin-positive area relative to islet cross-sectional area did not differ among the four experimental groups (Fig. 10H).

### Hepatic gene expression and insulin signaling

**Figure 11:**
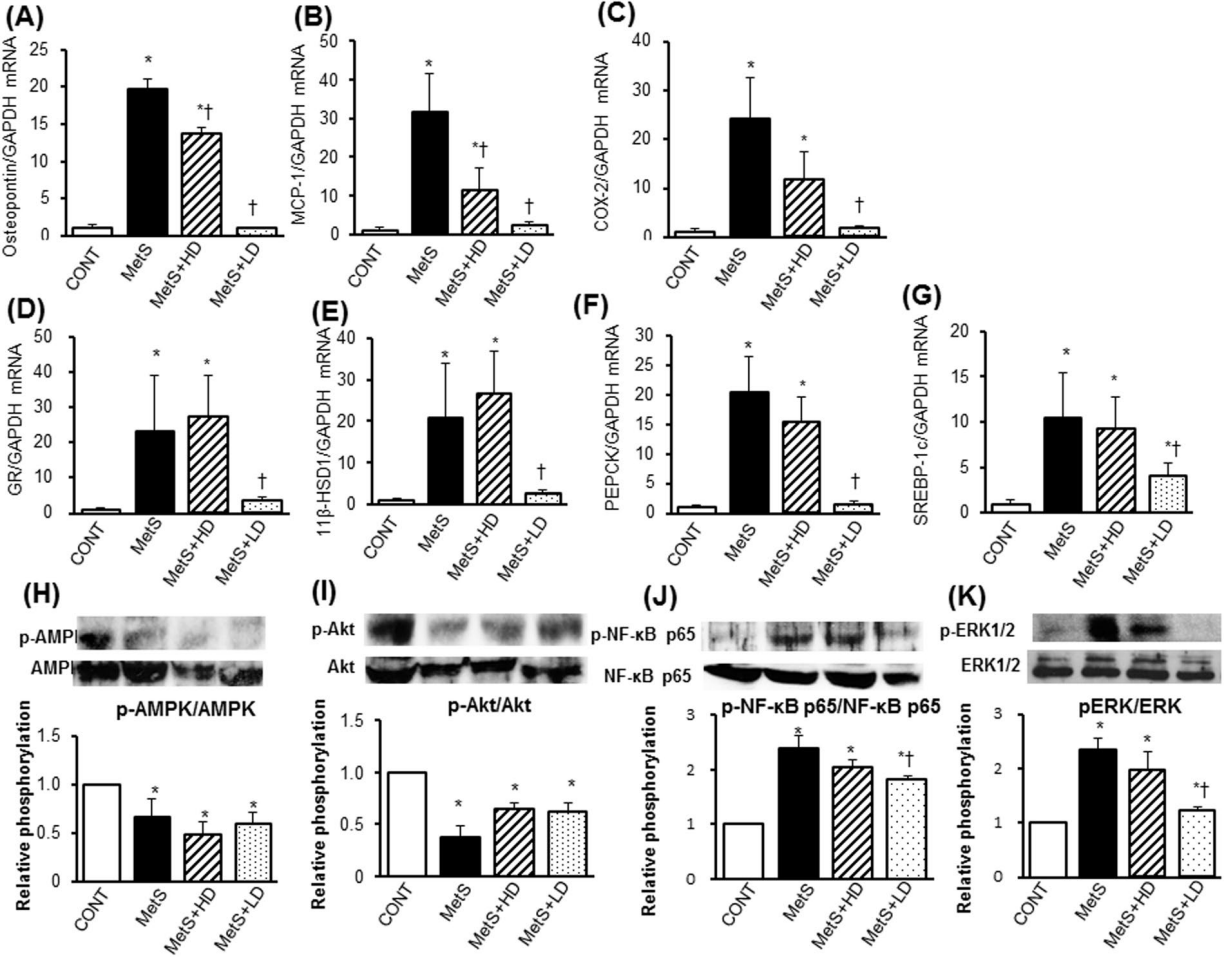
Effects of HK L-137 on hepatic gene expression and insulin signaling. (**A**–**G**) Quantitative RT-PCR analysis of relative osteopontin (**A**), MCP-1 (**B**), COX-2 (**C**), GR (**D**), 11β-HSD1 (**E**), PEPCK (**F**), and SREBP-1c (**G**) mRNA abundance in the liver. (**H**,**K**) Representative immunoblot analysis and densitometric quantification of phosphorylated (p−) and total forms of AMPK (**H**), Akt (**I**), NF-κB p65 (**J**), and ERK1/2 (**K**) in the liver. Data are means ± SEM (*n* = 5, 5, 7, and 7 for CONT, MetS, MetS + HD, and MetS + LD groups, respectively). *P < 0.05 versus CONT, ^†^*P* < 0.05 versus MetS.

To determine the mechanism of improved insulin sensitivity with HK L-137, we evaluated expression of genes related to inflammation, gluconeogenesis, and insulin signaling in hepatic tissue. Expression of osteopontin, MCP-1, and COX-2 genes in the liver was increased for the MetS group compared with the CONT group, and these increases were blocked by the low dose of HK L-137, with those in the expression of osteopontin and MCP-1 genes also being inhibited by the high dose (Fig. 11A–C). The amounts of glucocorticoid receptor (GR), 11β-hydroxysteroid dehydrogenase type 1 (11β-HSD1), phosphoenolpyruvate carboxykinase (PEPCK), and SREBP-1c mRNAs were increased in the liver of the MetS group in a manner sensitive to inhibition by the low dose of HK L-137 (Fig. 11D–G). Phosphorylation of AMPK and Akt was reduced in the liver of MetS rats and was not affected by HK L-137 (Fig. 11H,I). By contrast, HK L-137 at low dose suppressed the up-regulation of NF-κB p65 subunit and ERK1/2 phosphorylations in the MetS group (Fig. 11J,K).

## Discussion

We have here shown that treatment of DS/obese rats for 4 weeks with HK L-137 did not affect body weight gain or hypertension but ameliorated LV inflammation and fibrosis as well as attenuated adipocyte hypertrophy and inflammation in both visceral and subcutaneous adipose tissue. The low dose of HK L-137 in particular improved systemic inflammation and LV diastolic function, reduced subcutaneous fat mass, as well as attenuated systemic insulin resistance and improved insulin signaling in both visceral and subcutaneous adipose tissue. In addition, HK L-137 attenuated up-regulation of the lipogenic transcription factor SREBP-1c in visceral and subcutaneous adipose tissue as well as up-regulated IFN-β gene expression and increased the percentage of Treg cells in the spleen.

Increased oxidative stress is associated with hypertension, cardiac hypertrophy, and heart failure. Treatment with the low or high dose of HK L-137 suppressed LV oxidative stress, inflammation, and fibrosis, without affecting hypertension or LV hypertrophy, in DS/obese rats. In addition, the low dose of HK L-137 ameliorated LV diastolic dysfunction. The lack of effect of HK L-137 on LV hypertrophy and hypertension is consistent with the notion that cardiac hypertrophy is primarily load dependent^24^. Cardiac fibrosis is a pathological feature associated with hypertension and gives rise to LV diastolic dysfunction, likely as a result of increased LV diastolic stiffness. However, formation of fibrous tissue has also been shown to be independent of blood pressure and cardiac hypertrophy^25^. Macrophage infiltration and inflammatory responses have been implicated in fibrosis associated with various pathological conditions^25^, and synthesis of collagen type I by cardiac fibroblasts is inhibited by antioxidants^26^. Our results thus suggest that the antioxidant and anti-inflammatory properties of HK L-137 are largely responsible for its inhibitory effect on cardiac fibrosis. Nuclear factor-erythroid-2-related factor 2 (Nrf2)-antioxidant response elements (AREs) signaling pathway is considered to play a critical role in the stress defense system against oxidative stress^27^. The mitogen-activated protein kinase (MAPK) pathway, including ERK, c-Jun NH2-terminal kinase (JNK), and p38, activates the Nrf2-ARE signaling pathway^28^. *Lactobacillus gasseri* SBT2055 (LG2055) activated the Nrf2-ARE signaling pathway though activation of JNK, thus strengthening the defense system against oxidative stress^29^. LG2055 was used as heat-killed bacterial bodies and showed protective effects against oxidative stress, suggesting that some active substances likely contributed to the anti-oxidative effects of LG2055. In spontaneously hypertensive rats, *Lactobacillus fermentum* reduced aortic expression of Toll-like receptor 4 (TLR4) gene as well as exerted cardiovascular protective effects related to the improvement of vascular pro-oxidative and pro-inflammatory status^30^. Activation of TLR4 signaling results in increased NADPH oxidase-dependent superoxide production and inflammation in the vasculature. It is thus possible that HK L-137 may attenuate LV oxidative stress and inflammation in MetS rats though activation of Nrf2-ARE signaling and/or inhibition of TLR4 signaling.

The serine-threonine protein kinase Akt is an important mediator of phosphatidylinositol 3-kinase (PI3K) signaling, which is implicated in the regulation of cardiac growth and function^31^. Although Akt is required for physiological cardiac growth, long-term activation of Akt in Akt transgenic mice results in cardiac hypertrophy associated with pathological remodeling and cardiac dysfunction^32^. In addition, chronic pressure overload induced hepatic insulin resistance and increased circulating insulin levels in mice^33^, consistent with our previous results with DS/obese rats^34^. We have now shown that the low dose of HK L-137 alleviated LV diastolic dysfunction and reduced serum insulin levels, without affecting hypertension, LV hypertrophy, or cardiac Akt activity. These data are consistent with the previous findings that excessive cardiac insulin signaling results in exacerbation of cardiac dysfunction induced by pressure overload in mice, and that attenuation of hyperinsulinemia resulted in a substantial amelioration of overload-induced cardiac dysfunction^33^.

Oral administration of HK L-137 increased serum levels of IFN-β in healthy humans and IFN-β mRNA levels in the whole blood cells of pigs^17^ as well as induced an appreciable level of IFN-β in serum of a mouse model of influenza virus infection^19^. Moreover, *Tetragenococcus halophilus* strain KK221, a heat-killed lactic acid bacteria (LAB), induced IFN-β in a TLR3-dependent manner and suppressed expression of genes encoding inflammatory mediators such as TNF-α and IL-6 as well as alleviated colonic inflammation in a dextran sodium sulfate-induced colitis model^9^. A previous study reported that type 1 IFN is required for LPS-induced IL-10 production^35^. It is thus possible that IFN-β-mediated IL-10 production may be involved in the anti-inflammatory effects of LAB. Indeed, HK L-137, in particular at low dose, reversed or tended to reverse the down-regulation of IL-10 gene expression in LV, epididymal or subcutaneous fat tissues. Thus, we speculate that HK L-137 induced IFN-β in a TLR3-dependent manner, contributing to anti-inflammatory effects and protective immune responses.

NLRP3 inflammasome is associated with onset and progression of various diseases, including metabolic disorders. This complex contributes to the production and secretion of the mature IL-1β by catalyzing the conversion of procaspase-1 to caspase-1. Also, type 1 IFNs, including IFN-α and IFN-β, are NLRP3-specific inhibitors of inflammazome^36^. In this study, IFN-β gene expression was down-regulated in LV, splenic, epididymal and subcutaneous fat tissues of MetS rats and these effects were reversed by HK L-137 at low dose. In contrast, expression of IL-1β gene in these tissues were upregulated in a manner sensitive to HK L-137. Serum levels of mature IL-1β were also increased in MetS rats and this effect was alleviated by the low dose of HK L-137. These data suggest that the anti-inflammatory effects of HK L-137 may be mediated through IFN-β-induced inhibition of NLRP3 inflammasome activity.

CD4^+^ helper T lymphocytes are classified as Th1 cells, which produce IFN-γ and TNF-α, or as Th2 cells, which synthesize IL-4, IL-6, and IL-10^37,38^. Th1 cells mediate cellular immunity^39^, whereas Th2 cells promote antibody production^40^. The balance between Th1 and Th2 cells in an immune response is regulated by positive and negative interactions within and between the two types of cells^41^ and is an important determinant of various pathological conditions. HK L-137 induces IL-12 secretion by macrophages in healthy humans^17,19^ and triggers a Th1-type immune response^16–21^. However, we found that HK L-137 had no effect on the Th1/Th2 cell ratio in the spleen or adipose tissue of DS/obese rats. In addition, expression of IL-12 gene was up-regulated in LV, epididymal and subcutaneous fat, and splenic tissues of MetS rats and all of these effects were attenuated by HK L-137. IL-12 production is under positive and negative control by Th1 and Th2 cytokines, respectively. IFN-γ increases, whereas IL-10, IL-4, TGF-β, and IFN-α/β suppress IL-12 production^42^. IL-12 production is normally kept under inhibitory control by Th2 cytokines. Our results are thus in agreement with a previous study demonstrating that IFN-β-1b inhibits inducible IL-12 production in human peripheral blood mononuclear cells in an IL-10-dependent mechanism^43^. Therefore, the increased expression of IFN-β gene by HK L-137 may have been responsible for the down-regulation of IL-12 mRNA levels in LV, splenic, and adipose tissues of MetS rats. Whereas previous studies that detected an increase in the number of Th1 cells in response to HK L-137 treatment focused on acute inflammation such as that associated with influenza virus infection, the effect of HK L-137 on immune function in chronic inflammation such as that associated with metabolic disorders has remained unknown. It is thus possible that HK L-137 has differential effects on acute and chronic inflammation.

The NF-κB pathway plays an important role in the TLR4, an essential receptor for the recognition of LPS, -mediated immunomodulatory effect^44^. TNF-α derived from macrophages induces production of proinflammatory cytokines in adipocytes through activation of the NF-κB pathway as well as promotes adipocyte lipolysis through activation of the MAPK pathway^45^. Thus, saturated fatty acids released in large quantities from hypertrophied adipocytes likely induce the inflammatory changes locally in obese adipose tissue and systemically in circulating monocytes and/or macrophages infiltrated into other tissues through the TLR4/NF-κB pathway. In this study, the phosphorylation of both NF-κB p65 subunit and ERK1/2 were increased in LV, epididymal and subcutaneous fat, and hepatic tissues of MetS rats. Treatment with HK-L137, especially at low dose, attenuated activation of NF-κB and ERK in epididymal and subcutaneous fat and hepatic tissues. In LV tissue, HK L-137 also tended to reduce NF-κB activity as well as significantly attenuated ERK activation. These data suggest that HK L-137 prevented obesity-associated inflammatory responses in LV, adipose, and hepatic tissues through inhibition of the NF-κB and MAPK pathways.

Treg cells, which release anti-inflammatory cytokines such as IL-10, play an important role in restraining tissue inflammation^46^. Depletion of these cells has been associated with obesity and adipose tissue inflammation^47^. In contrast, we found that the proportion of Treg cells in the spleen or adipose tissue of DS/obese rats did not differ from that in DS/lean rats. However, the number of splenic Treg cells in DS/obese rats was increased by treatment with HK L-137 at the low or high dose, and the extent of macrophage infiltration in the left ventricle as well as in epididymal and subcutaneous adipose tissue in these animals was suppressed by administration of HK L-137. The increase in the number of Treg cells induced by HK L-137 in the spleen may thus have contributed to inhibition of inflammatory responses in these tissues. Furthermore, comparison of the extent of macrophage infiltration between epididymal and subcutaneous adipose tissue raises the possibility that more Treg cells moved from the spleen to subcutaneous adipose tissue than to epididymal fat in response to HK L-137 treatment.

TGF-β regulates peripheral T cell homeostasis and differentiation during the immune response^48,49^. TGF-β alone induces Foxp3 expression and Treg cell differentiation from CD4^+^ T cells whereas TGF-β in the presence of IL-6 inhibited Treg cell generation and diverted T cell differentiation to Th17 cells. HK L-137 attenuated the up-regulation of IL-6 and TGF-β mRNA expression in the spleen. Also, HK L-137 at low dose ameliorated an elevation of serum IL-6 levels in DS/obese rats, consistent with a previous result indicating that *Lactobacillus acidophilus* NCFM may diminish the translocation of lipopolysaccharide from the gut to the systemic circulation, thereby reducing the concomitant induction of proinflammatory cytokines through TLR4 signaling^50^. The reduction of IL-6 levels by HK L-137 may thus have contributed to the induction of Treg cells in the spleen. In addition, oral administration of heat-killed KK221 for 14 days up-regulated splenic IFN-β mRNA levels in wild-type mice^9^. It is thus likely that the HK L-137-induced reduction in IL-6 levels contributed to up-regulation of IFN-β gene expression in LV, epididymal and subcutaneous fat, and splenic tissues, thereby leading to an increase in splenic Treg cells in DS/obese rats. HK L-137 did not affect the number of Treg cells but increased IL-10 mRNA levels in epididymal fat tissue. Additionally, the low dose of HK L-137 tended to upregulate IL-10 mRNA expression in LV and subcutaneous fat tissue. We thus speculate that enhanced function of Treg cells, rather than Treg cell numbers per se, with HK L-137 likely contributed to inhibition of inflammatory responses in these tissues.

The RAAS contributes to metabolic disturbances associated with obesity^51^ as well as plays a crucial role in the T cell response during inflammation. Given that angiotensin II (Ang II) directly induces monocyte accumulation in bone marrow and the spleen, an increase in the number of pro-inflammatory cells triggered by activation of the RAAS may facilitate the progression of vascular inflammation and hypertension-associated cardiovascular disease as well as enhance oxidative stress and chronic inflammatory responses^52^. The Ang II/Ang II type 1 receptor axis contributes to the physiologic regulation of naive T cell migration to the spleen^22^ and blocking Ang II production suppressed autoreactive Th1 and Th17 cells and promoted antigen-specific CD4^+^Foxp3^+^ Treg cells^23^. We found that expression of RAAS-related genes was up-regulated in the spleen of MetS rats and that this up-regulation was attenuated by the low dose of HK L-137. In addition, HK L-137 induced Treg cells in the spleen whereas it did not affect the Th1/Th2 cell ratio in either the spleen or adipose tissue. The increase in the circulating level of IL-6 and IL-1β apparent in MetS rats was also inhibited by the low dose of HK L-137, suggesting that systemic inflammation was attenuated by this agent. These results suggest that HK L-137 may have suppressed the release of inflammatory monocytes-macrophages from the spleen via inhibition of the RAAS and enhancement of Treg cell redistribution, thereby contributing to down-regulation of cardiac and adipose tissue inflammation.

Inflammatory cytokines activate SREBP-1c, a key transcription factor for genes related to lipid synthesis^53^. Insulin also increases the expression of SREBP-1c in adipocytes, and Akt stimulates both SREBP-1c expression and lipogenesis^54^. Expression of SREBP-1c is up-regulated in obese or diabetic individuals as well as in animal models of these conditions^54^. Consistent with these previous observations, we found that the expression of TNF-α and SREBP-1c genes was increased in adipose tissue of DS/obese rats. Suppression of adipocyte enlargement in both epididymal and subcutaneous adipose tissue by HK L-137 may have resulted from a reduction in lipogenesis and fatty acid synthesis due to inhibition of SREBP-1c expression. The anti-inflammatory effects of HK L-137 were likely mediated by normalization of adipocyte size.

Our ITT data indicated that the low dose of HK L-137 attenuated the development of obesity-induced insulin resistance in DS/obese rats. Increased Akt phosphorylation in both visceral and subcutaneous adipose tissue may have contributed to this beneficial effect on systemic insulin sensitivity. Adiponectin inhibits mTOR/p70S6 kinase pathway by activation of AMPK, thereby contributing to Akt activation and improved insulin resistance. However, given that HK L-137 did not affect the serum adiponectin level or adiponectin mRNA abundance in visceral adipose tissue, adiponectin may not be responsible for the amelioration of insulin resistance by the low dose of HK L-137. The reduction in HOMA-β and total islet area per pancreas induced by the low dose of HK L-137 also suggest that the improved insulin sensitivity reduced the overload on pancreatic β cells and thereby contributed to a decrease in basal insulin secretion.

The low dose of HK L-137 prevented the MetS-associated increase in adipocyte size more effectively than did the high dose and improved insulin resistance. Although the mechanism for the greater beneficial effects of the low dose remains unclear, it is shown that HK L-137 promotes IFN-β production via pattern recognition receptors expressed by innate immune cells such as macrophages^9^. Scavenger receptor A (SR-A)- and CD36-mediated phagocytosis is responsible for the potent ability of HK L-137 to induce IL-12 p40^15^. Since serum lipid levels (especially LDL-cholesterol) were increased in DS/obese rats, SR-A and CD36, which are also receptors for oxidized LDL^55,56^, may be dysfunctional and SR-A- and CD36-mediated phagocytosis of HK L-137 could be reduced in these rats. It is thus possible that at low dose of HK L-137, SR-A- and CD36-mediated phagocytosis became predominant and the anti-inflammatory and protective immune responses by increased IFN-β were induced whereas, at high dose, other receptors (e.g. TLR2 or 4) were also activated and not only IFN-β but also proinflammatory cytokines were induced in these rats.

The intestinal microbiota and some probiotics are known to interact with the host’s immune system, thereby influencing both health status and disease risk. Recently, non-viable microbes such as HK L-137 have also been regarded as probiotics since they exhibited the beneficial effects equal to live microbes^8^. Unfortunately, we have no data on the effects of HK-L137 on intestinal microbiota in this model of MetS. However, a previous study with broiler chickens suggested that HK L-137 might activate intestinal function by increasing segmental filamentous bacteria^57^. It is thus possible that HK L-137 might affect intestinal microbiota, thereby altering the immune system in the gut.

In conclusion, short-term administration of HK L-137 had no effect on body weight gain or hypertension but reduced LV inflammation and fibrosis as well as adipocyte hypertrophy and inflammation in visceral and subcutaneous adipose tissue of DS/obese rats. In particular, HK L-137 at a low dose ameliorated systemic inflammation, LV diastolic dysfunction, and an increase in subcutaneous fat mass as well as improved systemic insulin sensitivity and insulin signaling in both visceral and subcutaneous adipose tissue. Our results suggest that the beneficial effects of HK L-137 on the heart and adipose tissue are related, at least in part, to suppression of systemic inflammation associated with up-regulation of IFN-β gene expression and an increase in Treg cells in the spleen. Further studies are thus warranted to investigate the potential application of HK L-137 to the prevention or treatment of metabolic disorders as well as to clarify the molecular mechanisms of its effects.

## Methods

### Animals

All animal experiments were approved by the Animal Experiment Co9mmittee of Nagoya University Graduate School of Medicine (Daiko district, approval nos. 028–039 and 029–013) and were conducted in accordance with the guidelines of Nagoya University Graduate School of Medicine as well as with the Guide for the Care and Use of Laboratory Animals (U.S. National Institutes of Health publication no. 85–23, revised 2011). Male inbred DS/obese rats were randomized to receive treatment with vehicle (MetS group, *n* = 8) or with HK L-137 (House Wellness Foods Corporation, Hyogo, Japan) at either a low (2 mg/kg) dose (MetS + LD group, *n* = 12) or a high (75 mg/kg) dose (MetS + HD group, *n* = 12) from 9 to 13 weeks of age. The doses of HK L-137 were determined on the basis of the results of previous studies and our preliminary observations^16,18,19,21^. HK L-137 was administered orally once daily via a gastric tube. Age-matched male homozygous lean littermates of DS/obese rats—DahlS.Z-*Lepr*^+^/*Lepr*^+^ (DS/lean) rats—served as control animals (CONT group, *n* = 8). All animals were fed normal laboratory chow containing 0.36% NaCl, and both the diet and tap water were provided ad libitum. SBP was measured weekly by tail-cuff plethysmography (BP-98A; Softron, Tokyo, Japan)^58^. The heart, liver, kidneys, spleen, visceral (retroperitoneal, epididymal, and mesenteric) and subcutaneous (inguinal) fat, and interscapular BAT were excised from the animals at 13 weeks of age after intraperitoneal injection of an overdose of sodium pentobarbital (50 mg/kg).

### Insulin sensitivity

Rats were subjected to an ITT at 13 weeks of age as described previously^58^. The HOMA-β index was calculated from fasting glucose and insulin concentrations also as previously described^59^.

### Cardiac function

At 13 weeks of age, rats were anesthetized with ketamine (50 mg/kg) and xylazine (10 mg/kg) and were subjected to transthoracic echocardiography and cardiac catheterization as described previously^60–62^.

### Serum and urine analysis

At 13 weeks of age, rats were placed in metabolic cages for collection of 24-h urine samples and determination of urinary protein and creatinine clearance. Blood was also drawn from the right carotid artery after anesthetization by intraperitoneal injection of sodium pentobarbital (50 mg/kg). The concentrations of total cholesterol, LDL–cholesterol, HDL–cholesterol, triglyceride, and free fatty acids in serum as well as those of creatinine in both serum and urine were measured by routine enzymatic assays^21^. Those of insulin (Morinaga Bioscience Institute, Yokohama, Japan), adiponectin (Otsuka Pharmaceutical Co., Ltd., Tokyo, Japan), IL-6 (R&D Systems, Inc., Minneapolis, MN, USA), and IL-1β (R&D Systems, Inc., Minneapolis, MN, USA) in serum were measured with enzyme-linked immunosorbent assay kits for the rat proteins.

### Histology

LV, visceral (epididymal) and subcutaneous (inguinal) fat, as well as pancreatic tissue was fixed, dehydrated, and embedded in paraffin. Sections were subjected to hematoxylin-eosin (H&E) staining in order to visualize individual cardiomyocytes and fat droplets as well as to Azan-Mallory staining in order to visualize collagen as a measure of cardiac fibrosis^60,62^. For visualization of macrophages or pancreatic β cells, sections were subjected to immunohistochemical staining with antibodies to rat CD68 (clone ED1; Chemicon, Temecula, CA, USA) or to insulin (Cell Signaling Technology, Beverly, MA, USA), respectively. Immune complexes were detected with biotin-conjugated secondary antibodies (Kirkegaard & Perry Laboratories, Gaithersburg, MD, USA) and the use of an ECL kit.

### Measurement of oxidative stress

NADPH oxidase activity in frozen LV tissue was measured with a lucigenin-enhanced chemiluminescence assay as previously described^63^. Dihydroethidium (DHE) staining was applied to evaluate superoxide levels in LV tissue sections also as described^21,64^.

### Quantitative RT-PCR analysis

Total RNA was isolated from LV, fat, hepatic, or splenic tissue and subjected to reverse transcription (RT) as described previously^35,59^. The resulting cDNA was subjected to real-time polymerase chain reaction (PCR) analysis with SYBR Mix Ex Taq II (Takara, Shiga, Japan) and specific primers also as described^59^. Primer sequences for ANP^60^, BNP^60^, collagen type I^65^ or III^65^, CTGF^64^, COX-2^64^, MCP-1^64^, osteopontin^64^, TNF-α^21^, IL-12 (AF177031.1), IL-1β (NM_031512.2), IL-10 (NM_012854.2), IFN-β1 (NM_019127.1), TGF-β^60^, GR^59^, 11β-HSD1^59^, PEPCK (NM_198780.3), SREBP-1c (NM_001276708.1), ACE^60^, AT_1A_ receptor^60^, MR^21^, Sgk1^21^, adiponectin^66^, and the p22^phox^ and gp91^phox^ subunits of NADPH oxidase^67^ were described previously. The mRNA abundance for target genes was normalized by that for the glyceraldehyde-3-phosphate dehydrogenase (GAPDH) gene.

### Immunoblot analysis

Proteins were isolated from LV, fat, or hepatic tissue and were subjected to immunoblot analysis as previously described^68–70^ with primary antibodies to the Thr^172^-phosphorylated or total forms of the α subunits of AMPK (Cell Signaling Technology, Beverly, MA, USA), to the Ser^473^-phosphorylated or total forms of the protein kinase Akt (Cell Signaling Technology), to the Ser^536^-phosphorylated or total forms of the p65 subunit of NF-κB (Cell Signaling Technology), or to the phosphorylated or total forms of ERK1/ERK2 (Cell Signaling Technology).

### Flow cytometry

Three-color flow cytometric analysis was performed as described previously^71^ with a FACSCalibur instrument (BD Biosciences, San Diego, CA, USA) and the following antibodies (BD Biosciences): fluorescein isothiocyanate–conjugated antibodies to CD4, peridinin chlorophyll protein–conjugated anti-CD25, phycoerythrin (PE)- and Cy5-conjugated anti-IL-4, PE-conjugated anti–interferon-γ (IFN-γ), and PE-conjugated anti-Foxp3. For Foxp3 staining, cells were first permeabilized with the use of a Cell Fixation/Permeabilization Kit (eBioscience, San Diego, CA, USA).

To identify Treg cells, the lymphocyte population was defined initially in a forward (FSC) and side scatter (SSC) gate. A second gate (logical) was then created around the CD4^+^ T cells. The gated cells were then analyzed for CD25 and Foxp3 expression, and the expression of CD25 and Foxp3 on CD4^+^ cells was determined as Treg cells. Likewise, to determine Th1 and Th2 cells, the lymphocyte population was also gated based on the scatter plot of FSC and SSC. Th1 and Th2 cells were identified as those that are CD4^+^ (y axis) and IFN-γ^+^ (x axis) and CD4^+^ (y axis) and IL-4^+^ (x axis), respectively.

### Statistical analysis

Data are presented as means ± SEM. One-way factorial analysis of variance (ANOVA) and Fisher’s multiple-comparison test were applied to evaluate differences among groups of rats at age 13 weeks. Time courses of parameters were compared among groups with two-way repeated-measures ANOVA. *P* values of < 0.05 were considered significant.

### Data availability

The datasets generated during and/or analyzed during the current study are available from the corresponding author on reasonable request.
